# Investigating Subjective Experience and the Influence of Weather Among Individuals With Fibromyalgia: A Content Analysis of Twitter

**DOI:** 10.2196/publichealth.6344

**Published:** 2017-01-19

**Authors:** Pari Delir Haghighi, Yong-Bin Kang, Rachelle Buchbinder, Frada Burstein, Samuel Whittle

**Affiliations:** ^1^ Faculty of Information Technology Monash University Melbourne Australia; ^2^ Monash Department of Clinical Epidemiology Monash University Melbourne Australia; ^3^ The Queen Elizabeth Hospital Adelaide Australia

**Keywords:** fibromyalgia, Twitter messaging, social networks, pain, weather, sentiment analysis, infodemiology

## Abstract

**Background:**

Little is understood about the determinants of symptom expression in individuals with fibromyalgia syndrome (FMS). While individuals with FMS often report environmental influences, including weather events, on their symptom severity, a consistent effect of specific weather conditions on FMS symptoms has yet to be demonstrated. Content analysis of a large number of messages by individuals with FMS on Twitter can provide valuable insights into variation in the fibromyalgia experience from a first-person perspective.

**Objective:**

The objective of our study was to use content analysis of tweets to investigate the association between weather conditions and fibromyalgia symptoms among individuals who tweet about fibromyalgia. Our second objective was to gain insight into how Twitter is used as a form of communication and expression by individuals with fibromyalgia and to explore and uncover thematic clusters and communities related to weather.

**Methods:**

Computerized sentiment analysis was performed to measure the association between negative sentiment scores (indicative of severe symptoms such as pain) and coincident environmental variables. Date, time, and location data for each individual tweet were used to identify corresponding climate data (such as temperature). We used graph analysis to investigate the frequency and distribution of domain-related terms exchanged in Twitter and their association strengths. A community detection algorithm was applied to partition the graph and detect different communities.

**Results:**

We analyzed 140,432 tweets related to fibromyalgia from 2008 to 2014. There was a very weak positive correlation between humidity and negative sentiment scores (*r*=.009, *P*=.001). There was no significant correlation between other environmental variables and negative sentiment scores. The graph analysis showed that “pain” and “chronicpain” were the most frequently used terms. The Louvain method identified 6 communities. Community 1 was related to feelings and symptoms at the time (subjective experience). It also included a list of weather-related terms such as “weather,” “cold,” and “rain.”

**Conclusions:**

According to our results, a uniform causal effect of weather variation on fibromyalgia symptoms at the group level remains unlikely. Any impact of weather on fibromyalgia symptoms may vary geographically or at an individual level. Future work will further explore geographic variation and interactions focusing on individual pain trajectories over time.

## Introduction

Fibromyalgia syndrome (FMS) is a chronic rheumatic syndrome of unknown etiology, characterized by persistent widespread musculoskeletal pain and stiffness and a variety of other symptoms including fatigue, poor sleep quality, altered cognition, and affective dysfunction [1]. Fibromyalgia symptoms can worsen or flare up over time. Individuals with FMS frequently attribute variation in their symptoms to environmental factors, including the weather [2-8]. Investigating the relationship between weather and FMS symptoms can be considered a key step toward a systematic study of flare-ups [3]. Such studies will allow the development of theories about the causes and possible approaches to treatment of FMS symptoms that can be tested empirically in future. A good understanding of the influence that weather conditions could have on FMS symptoms will provide clinicians with valuable insights into the context in which pain occurs and the association of these environmental factors (eg, temperature and humidity) with pain. The results can potentially provide the basis for developing more effective treatment methods, contributing to improving the quality of life of large numbers of patients.

Although existing studies have suggested an influence of weather on the pain experienced in FMS and related conditions [2-7], no consistent effect of a particular weather condition on FMS symptoms has been demonstrated. Such inconsistency may reflect individual sensitivity to weather conditions [2] or the complex interplay of other mediating factors such as low mood and lack of sleep or exercise [9]. Alternatively, the limitations of traditional data collection techniques such as questionnaires [2], diaries [5], and open-ended questions [3] may have led to a failure to detect a relationship between weather and symptoms in FMS. Such limitations may affect both internal validity (eg, response bias [2], small sample size [5]) and external validity (eg, sampling in particular geographic locations [3]).

Social networking sites introduce an unprecedented opportunity to collect first-person data from a very large population across diverse locations [10]. They provide a rich source of real-time data that can be collected and analyzed for health research including infodemiology and infoveillance [11,12]. Among existing sites, Twitter has been the most popular platform for accessing and analyzing infodemiology data [13,14]. Twitter provides an easier access to real-time and historical data through Twitter application program interfaces (APIs) and third-party tools, compared with the other platforms such as Facebook. Moreover, extensive studies have been conducted using Twitter that provide useful guidance.

In recent years, Twitter has been widely used as a platform to disseminate and share health-related information [15-20]. “Crowdsourced” data have been shown to be as reliable and viable as traditional survey data [21]. Sentiment analysis of a large number of messages can provide valuable insights into crowd mood [22] and health status [16,19].

Individuals with FMS are known to be active Internet users [23]. However, to date there has been relatively little research regarding the ways in which this community interacts via Twitter. Analysis of patterns of use within the FMS community may allow a deeper understanding of the subjective experience of this common pain syndrome and may inform future attempts to utilize social media as a therapeutic tool. We consider this form of analysis complementary to other forms of epidemiological inquiry.

In this infodemiology study, we used computerized content analysis of tweets to investigate the subjective experience of FMS in a large, widely distributed population and any association with coincident environmental factors. To better understand the environmental context around an individual’s symptoms and pain events, we were also interested to explore and uncover thematic clusters and communities. We hypothesized that detected communities identify specific contexts including environmental context such as weather. Toward this aim, we also performed graph analysis on tweets to understand the existence of specific topics associated with symptom severity and applied community detection to partition the graph and detect different communities. To the best of our knowledge, this is the first research attempt to study environmental associations of fibromyalgia pain using Twitter content analysis in a large sample of individuals with fibromyalgia.

## Methods

### Sample

We collected tweets from January 2008 to November 2014. The search used the keywords #fibromyalgia, #fibro, and #spoonie. The term “spoonie” is commonly used in tweets referring to the experience of chronic illnesses with prominent fatigue, particularly FMS but also illnesses such as chronic fatigue syndrome and systemic lupus erythematosus (SLE), and arises from the use of spoons as a metaphor for an individual’s ability to carry out daily tasks (“the spoon theory” [24]).

Tweets were collected during numerous search cycles programmatically using the Python language. Each cycle queried tweets for a 30-day period. The process involved a total of 84 cycles to retrieve the historical data over 7 years (2008-2014). Each cycle had multiple iterations to collect all the tweets because the API had a call limit of 100 tweets per request. We collected the data between November 11 and 24, 2014. The search was filtered for English-language tweets.

The date and time of posting of each tweet along with the location were also collected. This allowed us to collect the climate data for each tweet from the public Web APIs. Because of the large number of tweets, we wrote a Python program to collect the weather data. There was a limit on the number of calls per day (ie, a total of 1000 calls per day); therefore, it took November 2014 to February 2015 to collect the weather data for all the tweets. It is noteworthy that most of the location information collected was relative location such as city, state, or country. We excluded tweets that did not have any location data. The weather variables that we retrieved included temperature, humidity, wind speed, feels like, heat index, wind chill, and dew point. Because the date and time in our collected tweets from different locations were expressed in Greenwich Mean Time (GMT) or Coordinated Universal Time (UTC), we could directly use it in the weather API queries, which also support GMT and UTC.

### Ethics

Monash University’s Ethics Committee approved this study.

### Sentiment Analysis and Correlation Tests

To perform sentiment analysis, we incorporated the Stanford CoreNLP [25] libraries. The Stanford CoreNLP works by initiating the properties object and defining annotators [26]. The annotation object executes a number of predefined annotators such as tokenization, sentence splitting, POS (part-of-speech) tagging, and named entity recognition (NER) on text and retrieves an annotated document that can train the sentiment analyzer [26]. The Stanford CoreNLP also includes a sentiment tool and it can be used to analyze the text by adding “sentiment” to the list of annotators [26]. The classifier in CoreNLP is trained using a deep learning algorithm. In our analysis, the classifier assigned a positive score and a negative score between 0 and 100 to each tweet. The 2 assigned scores for each tweet added up to 100.

Sentiment analysis commonly uses a dictionary of opinion words (the “opinion lexicon”) to identify and determine sentiment orientation (positive, negative, or neutral). In the opinion rule examples [27], “pain” was considered a “negative” word in the opinion lexicon. The negative sentiment scores can therefore be used to represent severe symptoms such as the pain severity in an individual. In our research, we make the assumption that the severity of negative symptoms (particularly pain) in individuals with FMS can be estimated based on the negative and positive scores assigned to the tweets (a value between 0 and 100).

This means that a high negative sentiment score (and a low positive score) of a tweet will be interpreted as representing more severe pain and unpleasant physical sensations (eg, “too tired to write, too tired to function, just too tired. i ache all over. damn #fibromyalgia. maybe epsom salt bath...” with a negative score of 99.24 and a positive score of 0.76). On the other hand, a low negative score and a high positive score will imply a low severity of pain and related symptoms (eg, “Celebrating the very fact that I am up at 9:30 p.m. I lived through Saturday!#fibro” with a negative score of 0.07 and a positive score of 99.93).

In the second step, we used the Pearson correlation test to investigate the association between negative sentiment scores (as a measure of symptom severity) and climatic variables. We started with a null hypothesis that there is no correlation between FMS symptom severity and weather conditions. The correlation test was first conducted for all the tweets posted from all over the world. Considering that the weather conditions can vary considerably in different geographic locations (and climate zones), and individuals’ feelings and adaptation to the weather conditions may also vary by location, we also performed correlation tests for locations that had a total number of users greater than 1000. Statistical analysis was performed using IBM SPSS Statistics 23.0 (IBM Corporation).

### Graph Analysis and Community Identification

In addition to the correlation tests, we performed graph analysis on the tweet corpus to gain further insight into the common terms that are used by individuals with FMS and how these terms are associated with each other. The graph analysis identifies the most relevant and commonly used terms and their relationships to the domain. More specifically, our aim was to explore and use the following 4 measures.

#### Measure 1

The occurrence frequency distribution of terms relevant to the corpus.

#### Measure 2

The degree centrality (or local connectivity) of each of these terms measured by calculating the number of relations that the term has regardless of whether it is an in-relation or out-relation within the corpus. In addition, we also considered the betweenness centrality of terms in the graph. This metric measures all the shortest paths between every pair of terms in the graph and then counts how many times a term is on a shortest path between two others. In our context, it can detect the dominant terms that occupy a set of intermediate terms observed between two other terms.

#### Measure 3

The association strengths among the terms that are measured by calculating how frequently particular terms are associated together based on their co-occurrences within the corpus.

#### Measure 4

The community structures that determine the level of dissemination of content within the domain corpus.

The first measure was determined by counting the total occurrence frequency of each of the terms extracted from our corpus. The second and third measures were achieved using graph analysis. For this, we constructed a term-term association graph. The association strength between 2 terms was measured by how often they co-occur within the corpus.

The last measure was done by decomposing the term-term association graph into subgroups or communities, which are sets of highly interconnected terms. We used the Louvain method [28] as a community detection algorithm implemented in Gephi [29]. This method is simple and efficient and one of the most widely used methods for identifying communities in a large network [17]. The Louvain method is an iterative algorithm that scans step by step the density of connections between the nodes in the graph. The nodes that are more densely connected to one another are included in the same community. This allows the potential detection of a priori unknown communities of highly associated terms in the FMS domain.

### Preprocessing Steps

We first split each tweet in the corpus at whitespace characters to obtain individual terms [17]. We converted these terms to lowercase and removed stopwords (eg, common terms such as “the” and “is”), tweet-specific meta terms (eg, terms using “via” prefixes), symbols (eg, “@”), punctuation, and numbers. We then constructed a term-document matrix, where a row represents a tweet identifier and a column represents an individual term extracted from the corpus. By counting all the frequencies under each term within the matrix, we produced the frequency distribution of the terms. In order to restrict the size of the term-document matrix, we removed those terms whose sparse rates were greater than.999, that is, those terms that had a 99.9 sparse percentage of empty elements. A sparse percentage of 0.999 was chosen to ignore very infrequent terms and to retain only the terms whose document frequencies were greater than “N×(1−.999),” where N is the total number of tweets considered.

To measure the degree centrality of the terms and association strengths among the terms, we constructed a term-term graph. To build this graph, we created a term-term matrix, where rows and columns represent unique terms and a cell represents how many times a certain term co-occurs with another term.

## Results

### Correlation Tests

A total of 157,377 tweets were collected. After excluding the retweets, and tweets that contained a URL or did not have any location data, a total of 140,432 tweets were available for analysis. There was a very weak positive correlation between humidity and negative sentiment scores (*r*=.009, *P*=.001). There was no significant association between negative sentiment scores and other environmental variables (ie, temperature, wind speed, feels like, heat index, wind chill, and dew point).

There were 8 locations that had at least 1000 users, all of which were US states (California, Colorado, Florida, Georgia, Minnesota, New York, Ohio, and Texas). California had the highest number of tweets (5149) among all the locations.

There was no significant correlation between negative sentiment scores and any of the weather variables in Colorado, Florida, Georgia, Minnesota, Ohio, and Texas. However, the correlation tests for California and New York showed significant but weak relationships between negative sentiment scores and climatic variables (see Table 1). In California, there was a weak positive relationship between negative sentiment scores and humidity and a weak negative correlation between negative sentiment scores with temperature, feels like, heat index, and wind chill. In New York, there was only a weak positive relationship between negative sentiment scores and wind speed.

**Table 1:** Correlation between environmental variables and negative tweet sentiment, by US state.

Location		Temperature	Humidity	Wind speed	Feels like	Heat index	Wind chill	Dew point
**California (n=5149)**								
	Pearson *r*	−.062	.050	−.030	−.057	−.059	−.060	.005
	*P* value^a^	<.001	<.001	.03	<.001	<.001	<.001	.71
	Direction of relationship	Negative	Positive	Negative	Negative	Negative	Negative	Positive
**New York (n=2058)**								
	Pearson *r*	−.035	−.023	.078	−.039	−.036	−.037	−.055
	*P* value^a^	.11	.28	<.001	.07	.10	.09	.01
	Direction of relationship	Negative	Negative	Positive	Negative	Negative	Negative	Negative

^a^Statistical significance was defined as *P*<.001.

### Graph Analysis

From the 140,432 tweets in the corpus, we extracted 61,469 terms after taking the preprocessing steps. After removing the sparse terms, 1170 terms were finally used to create a term-term matrix.

The occurrence frequency distribution results showed that the 2 most frequent terms were “pain” (appearing 24,323 times) and “chronicpain” (19,523 times). Among the top 25 terms (Multimedia Appendix 1), 2 broad groupings of terms may be identified: those that relate to medical conditions or states (eg, “chronic,” rank=5; lupus, rank=8; sleep, rank=19) are mixed with nonphysical terms (eg, day, rank=3, and today, rank=6).

The degree centrality scores of the top 2 terms “pain” and “chronicpain” were 1073 and 1070, respectively (Multimedia Appendix 1). These data indicate that these pain-related terms are the most highly associated with other terms. The mean degree centrality of the 1170 terms was 549, meaning that each term co-occurred with 549 terms in the corpus, on average.

**Figure 1:**
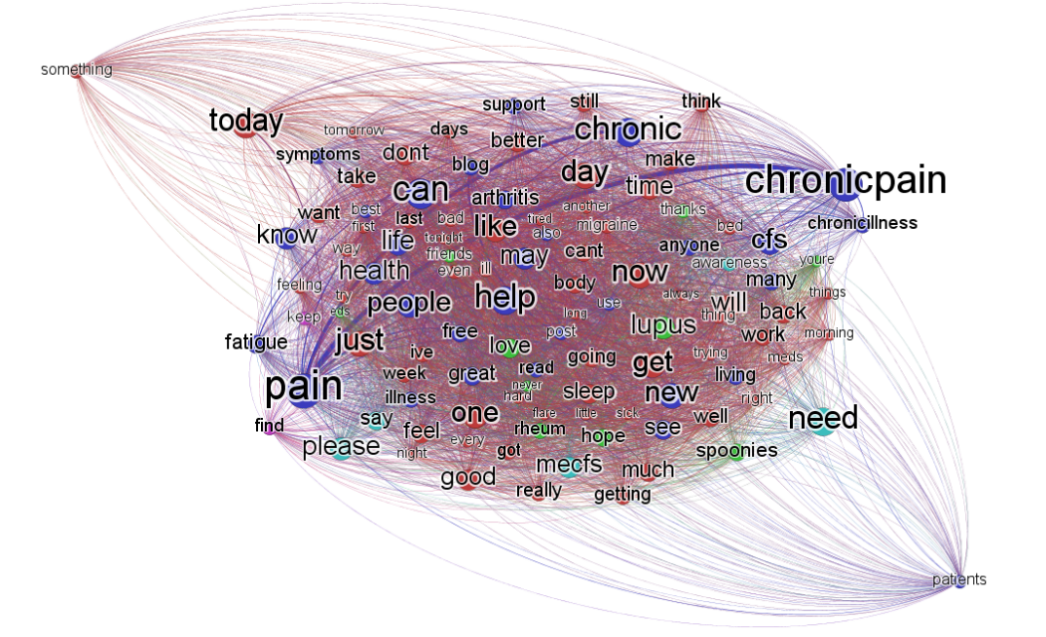
The term-term graph built based on betweenness centrality.

Figure 1 shows the term-term graph constructed based on betweenness centrality. Betweenness centrality measures the number of shortest paths from all nodes in a graph to all others that pass through that term, and it may be considered an indicator of the importance of a term. The more important terms are denoted in larger term labels according to betweenness centrality. We used the top 10% terms, chosen by the degree centrality scores, to make the graph more readable and highlight the terms with higher scores. Degree centrality of a term counts how many links the term has with other terms within the fibromyalgia tweet corpus. We observed that the top 10 terms according to betweenness centrality were “pain,” “chronicpain,” “can,” “chronic,” “help,” “need,” “today,” “day,” “now,” and “just.” The association strength between 2 terms “pain” and “chronicpain” was the highest, co-occurring 5385 times (Multimedia Appendix 1).

### Community Identification

Results of community detection using the Louvain method [28] showed that 2 communities (communities 1 and 3) had the highest number of terms (Multimedia Appendix 1). This may indicate that Web-based communications about fibromyalgia form a limited dissemination of the content mostly covered by 2 communities.

To investigate the term associations within the 2 largest communities, we generated associations of terms within each of these communities. Tables 2 and 3 show the top 15 associations of terms. In Tables 2 and 3, the “weight” indicates the number of co-occurrences of a pair of terms.

We also examined these communities for weather-related terms. Table 4 lists the weather-related terms identified in community 1. The weight of each term represents the number of associated terms (co-occurred terms) with a given term.

**Table 2:** The top 15 associations of terms within community 1.

Rank	Term 1	Term 2	Weight
1	Like	Feel	1133
2	Today	Day	1048
3	Just	Day	869
4	Raw	Healed	747
5	Little	Day	694
6	Good	Day	678
7	Day	Bad	551
8	Right	Now	550
9	Night	Last	500
10	One	Day	479
11	Sleep	Night	464
12	Feel	Better	463
13	Get	Day	462
14	Like	Day	429
15	Like	Just	426

**Table 3:** The top 15 associations of terms within community 3.

Rank	Term 1	Term 2	Weight
1	Pain	Chronicpain	5385
2	Pain	Chronic	4116
3	Post	Blog	2428
4	Day	Awareness	1498
5	Pain	Can	1456
6	Pain	Free	1348
7	Pain	Day	1311
8	Illness	Chronic	1304
9	Fatigue	Chronic	1249
10	Chronicpain	Can	1228
11	Keep	Fit	1190
12	Free	Chronicpain	1144
13	Like	Feel	1133
14	Pain	Back	1130
15	Today	Pain	1051

**Table 4:** The weather-related terms within community 1.

Terms	Weight
Weather	772
Cold	749
Hot	668
Rain	584
Warm	509
Sun	443
Winter	397
Summer	359

## Discussion

### Principal Findings

The main findings of this study add to the existing evidence base that suggests that there is no single weather condition that has a uniform effect on symptom expression in individuals with FMS at the group level. Although a statistical correlation between humidity and symptom severity was found in the tweets posted worldwide, the strength of the correlation was of negligible explanatory value.

Only several US states provided enough tweets (ie, >1000) for analysis within discrete geographic areas. Although there is some global variation in FMS prevalence [30], it is likely that this finding reflects a higher proportion of active Twitter users in the United States. However, this requires further investigation.

The interpretation of significant correlation test results in Table 1 (although very weak) could be that, in California, when the temperature, feels like, or heat index increases, the symptom severity (pain) decreases; however, an increase in humidity induces an increase in symptom severity (pain). This result agrees with the study by Macfarlane et al [9] that suggests patients believe warmer and drier climates improve their symptoms. Yet, Table 1 shows a different result for New York. In New York, when the wind speed increases the symptom severity (pain) increases. This underlines the fact that any impact of weather variables such as temperature, humidity, and wind speed on fibromyalgia symptoms (if true) may vary by geographic location.

Each state represented a distinct climate condition that was important in our tests. Correlation patterns in these states varied. A clear association between weather variables and FMS symptoms also did not emerge from these data. This large dataset is further evidence that any associations between climatic variables and pain symptoms seen in single locations are unlikely to reliably represent causal influences and would not be expected to be replicated in larger samples.

We did not test for the influence of interactions between weather variables on FMS symptoms in this study, although a clinically significant effect of any such interaction would be unlikely. Furthermore, these data do not exclude an effect of weather on the FMS experience at the individual level, and it remains plausible that particular weather states (or changes in weather states) have different effects between individuals with FMS or within individuals over time.

The graph and community detection analyses revealed several distinct patterns of communication in the FMS community. Analysis of the top 25 term associations shows that the term “pain” is associated with 5 other terms, which creates a combination of pain-related concepts (ie, “pain,” “chronicpain,” “chronic”) and non–pain-related terms (ie, “can,” “free,” “day,” “back,” “today,” and “new”; Multimedia Appendix 1). The term “chronic” is also associated with other symptom-related terms (“pain,” “illness,” “fatigue,” and “syndrome”).

The term-term graph built based on betweenness centrality (Figure 1) showed that the top 10 terms according to betweenness centrality included a number of words that are used to describe time (eg, “today,” “day,” “just,” and “now”). This may reflect the use of Twitter as a description of recent or immediate events or subjective states. This can be considered as a reassuring finding that Twitter provides valuable insight into current symptoms, especially if we are correlating with concurrent environmental conditions. Examples of such tweets from our corpus include the following:

I am at -3 spoons right now at least. This sucks. #spoonie

I feel like I have half a spoon right now and I just had a nap. #spoonie

We also notice that the word “can” is identified within the top 10 and “can’t” has the 41st rank. These words may represent concepts of functional ability among individuals.

An example of a tweet containing the term “can”:

Waiting for the meds and sedative to kick in so I can go back to sleep. #spoonie

An example of a tweet that includes “can’t”:

Can't sleep because of my back. Just took a hot shower and it didn't help enough. 1 spoon left right now. #spoonie

We identified a total of 6 communities of communication patterns among tweets relating to FMS. This is considerably smaller than the number of communities detected within the domain of “pain” (ie, 161 communities) using the same community detection algorithm reported in a study by Tighe et al [17]. Of the 6 communities in our study, 2 accounted for the majority of terms (communities 1 and 3; Multimedia Appendix 1). It can be observed that community 1 mostly includes the terms about “subjective experience” that reflect a description of the current symptom state (eg, “now,” “feel,” “well,” “better”).

In contrast, the terms in community 3 tend to reference broader issues of diagnosis, community, support, and awareness (eg, “chronic,” “life,” “support,” “anyone”).

In this study, we hypothesized that community detection would identify specific weather-related terms. We were able to detect a number of weather-related terms in community 1 (see Table 4). Examples of these tweets include the following:

Of course, the rainy weather isn't helping. #spoonie #fibro

Its cold and rainy in N.C!!! #fibro kick'N now!!! YuK,YuK!!! But it won't wiN! I will!

Not liking this snow. body is revolting. pls send warm weather or send me somewhere warm. #fibro sux. should just go back 2 bed. #spoonie

One of the tweets about the impact of the weather was posted from Scotland (March 18, 2010):

People say the weather has no effect on #fibro. THEY LIE ITS RAINING AND I'M ACHING. Sorry just had to get that out

While the identified weather-related terms had a low degree (see Table 4), and some of these terms such as “warm” were used in other contexts (eg, “warm bath,” “warm clothes,” or “warm hugs”), the results show the potential of social media platforms for studying the impact of environmental factors on chronic diseases from a first-person perspective.

Given that the terms in communities 1 and 3 represented more than 80% of all terms in our corpus, it is likely that individuals who tweet about fibromyalgia predominantly use this social medium either as a means of immediate symptom expression or to access or develop a Web-based social structure for the purpose of interpersonal support, advice, and advocacy.

### Limitations

Our study has several limitations. First, one of the keywords that we used was “spoonie.” This is because our first attempt to collect tweets using only #fibro and #fibromyalgia returned a limited number of tweets. When we examined the tweets, we realized that most individuals with fibromyalgia use #spoonie to tag their tweets. The inclusion of the search term #spoonie substantially improved the sensitivity of our search but at the cost of some loss of specificity. There were some tweets that were posted by individuals who might have other chronic conditions. This is reflected in the inclusion of some overlapping data related to other chronic illnesses such as SLE and chronic fatigue syndrome.

Second, on many occasions, multiple hashtags occurred together in a single tweet. This may reflect the high incidence of “secondary” FMS in individuals with other rheumatic diseases. Examples include the following:

Felt a little discouraged today when my doctor thought it would take about a year for me to get back to where I was. #spoonie #lupus #fibro

Feeling horrible #Lupus, #Fibro, & now the FLU!! Ugh Going to the Dr on Wed Putting on headset & sleeping till Wed. lol

Third, our tests for US states used relative location information (states); however, it is unlikely that more precise location data would have substantially affected the results. We cannot exclude an effect on the results if the Twitter users spent most of their time indoors [2,5].

### Conclusions

Web-based social networking services enable users to publicly share their thoughts and opinions, including those related to health behavior. Twitter is one of the most widely used social networking and microblogging services. Tweets exist in the public domain and are associated with additional data including date and time, as well as location. Aggregation and sentiment analysis of a large number of messages can be used to better understand the effects of contextual variables such as weather on chronic diseases. It also introduces new possibilities of measuring safety and effectiveness of new treatments and vaccines in real time and over time through surveillance of public opinions on social media [31].

This study shows that computerized content analysis of social media data provides a novel and potentially powerful approach to understanding variation in the FMS experience from a first-person perspective. Yet, a uniform causal effect of weather variation on FMS symptoms at the group level remains unlikely. Any impact of weather on FMS symptoms may vary geographically or at an individual level. Future work will further explore geographic variation and interactions focusing on individual pain trajectories over different seasons. We also plan to extend our work on graph analysis and community identification by considering semantic relationships.

## Appendix

Multimedia Appendix 1 Terms frequency and degree centrality.

## Abbreviations

API: application program interface
FMS: fibromyalgia syndrome
SLE: systemic lupus erythematosus
