# Supplementary material for: Investigating Subjective Experience and the Influence of Weather Among Individuals With Fibromyalgia: A Content Analysis of Twitter
Source: JMIR Public Health Surveill. 2017 Jan 19;3(1):e4. doi: 10.2196/publichealth.6344 (PMC5290295; doi:10.2196/publichealth.6344)
Supplement: Multimedia Appendix 1 [file publichealth_v3i1e4_app1.pdf]

Supplementary Appendix

Table 1. The top-25 terms according to their occurrence frequencies in the tweet corpus

Rank	Term	Frequency
1	pain	24,323
2	chronicpain	19,523
3	day	8,389
4	can	7,104
5	chronic	6,951
6	today	6,764
7	just	6,246
8	lupus	5,500
9	get	5,489
10	new	5,349
11	now	5,180
12	help	5,064
13	like	4,992
14	blog	4,329
15	know	4,161
16	need	4,115
17	mecfs*	4,099
18	awareness	3,898
19	sleep	3,828
20	post	3,648
21	good	3,606
22	life	3,594
23	back	3,593
24	dont	3,593
25	time	3,489

*mecfs refers to Chronic Fatigue Syndrome (CFS) Myalgic Encephalomyelitis (ME)

Table 2. The top -25 terms according to their degree centrality

Rank	Term	Degree Centrality
1	Pain	1073
2	chronicpain	1070
3	Can	1068
4	Get	1068
5	Just	1067
6	new	1065
7	day	1063
8	like	1063
9	know	1062
10	today	1062
11	now	1060
12	need	1057
13	one	1055
14	help	1051
15	time	1051
16	lupus	1051
17	will	1050
18	good	1049
19	mecfs	1042
20	dont	1041
21	cfs*	1040
22	chronic	1034
23	life	1034
24	feel	1033
25	back	1031

*cfs refers to Chronic Fatigue Syndrome (CFS)

Table 3. The top-25 term associations according to their co-occurrences

Rank	Term 1	Term 2	Co-occurrence
1	pain	chronicpain	5385
2	pain	chronic	4116
3	post	blog	2428
4	day	awareness	1498
5	pain	can	1456
6	pain	free	1348
7	pain	day	1311
8	illness	chronic	1304
9	fatigue	chronic	1249
10	chronicpain	can	1228
11	keep	fit	1190
12	free	chronicpain	1144
13	like	feel	1133
14	pain	back	1133
15	today	pain	1051
16	help	chronicpain	1050
17	today	day	1048
18	syndrome	chronic	1025
19	chronicpain	awareness	1018
20	post	new	1016
21	syndrome	fatigue	1004
22	move	keep	997
23	pain	new	992
24	help	can	988
25	move	fit	985

Table 4. The top-25 terms detected with the two populous communities.

Community 1				Community 3	
Rank	Term 1	Degree		Term 1	Degree
1	get	1068		pain	1073
2	just	1067		chronicpain	1070
3	day	1063		can	1068
4	like	1063		new	1065
5	today	1062		know	1062
6	now	1060		help	1051
7	one	1055		cfs	1040
8	time	1051		life	1034
9	will	1050		chronic	1034
10	good	1049		people	1027
11	dont	1041		may	1014
12	feel	1033		see	1001
13	much	1031		great	996
14	back	1031		health	996
15	well	1025		anyone	974
16	really	1020		arthritis	968
17	going	1013		many	955
18	sleep	1013		fatigue	949
19	want	1011		chronicillness	944
20	think	1010		blog	944
21	cant	1010		living	920
22	take	1010		post	918
23	better	1007		also	914
24	make	1007		illness	913
25	work	1007		support	905
